# Supplementary material for: Avian malaria-mediated population decline of a widespread iconic bird species
Source: R Soc Open Sci. 2019 Jul 17;6(7):182197. doi: 10.1098/rsos.182197 (PMC6689627; doi:10.1098/rsos.182197)
Supplement: Supplementary material [file rsos182197supp1.docx]

**SUPPLEMENTARY MATERIAL**

**Table S1.** Annual counts of chirping male sparrows used to measure changes in local breeding populations between 2005 and 2009. Two visits were carried out annually between mid-March and mid-May. Visit n. = visit number. For legend to site code see Figure 1 and Table 2.

|  | **2005** | | **2006** | | **2007** | | **2008** | | **2009** | |
| --- | --- | --- | --- | --- | --- | --- | --- | --- | --- | --- |
| **Visit n.**  **Site code** | **1** | **2** | **1** | **2** | **1** | **2** | **1** | **2** | **1** | **2** |
| A | 22 | 44 | 29 | 26 | 24 | 8 | 10 | 17 | 7 | 33 |
| B | 14 | 21 | 15 | 21 | 22 | 16 | 12 | 20 | 27 | 43 |
| C | 14 | 26 | 6 | 11 | 18 | 15 | 7 | 20 | 18 | 28 |
| D | 19 | 23 | 1 | 15 | 11 | 18 | 28 | 20 | 12 | 15 |
| E | 38 | 38 | 28 | 38 | 17 | 47 | 39 | 30 | 24 | 16 |
| F | 2 | 6 | 3 | 3 | 2 | 5 | 4 | 4 | 10 | 9 |
| G | 10 | 9 | 7 | 6 | 12 | 5 | 8 | 11 | 3 | 5 |
| H | 56 | 35 | 17 | 19 | 16 | 28 | 25 | 36 | 12 | 14 |
| I | 14 | 24 | 21 | 20 | 20 | 11 | 23 | 34 | 20 | 34 |
| J | 32 | 44 | 52 | 28 | 19 | 23 | 10 | 24 | 28 | 25 |
| K | 34 | 26 | 15 | 23 | 21 | 29 | 29 | 21 | 6 | 25 |

**Table S2.** Outputs of candidate models for investigating house sparrow survival in relation to age of individual birds, site and year of ringing and resighting either varying between sites, years, ages or kept constant. K= number of parameters; *=interaction; Phi=survival; p=recapture rate.

| **Model** | | **K** | **AICc** | **ΔAICc** | **AICc Weights** | **Deviance** |
| --- | --- | --- | --- | --- | --- | --- |
| 1 | Phi(age + year)p( .) | 5 | 1169.648 | 0.000 | 0.271393 | 1159.559 |
| 2 | Phi(year)p( .) | 3 | 1171.034 | 1.386 | 0.135731 | 1164.999 |
| 3 | Phi(age + site + year)p( .) | 9 | 1171.856 | 2.207 | 0.090004 | 1153.587 |
| 4 | Phi(age + year)p(year) | 6 | 1171.905 | 2.257 | 0.087814 | 1159.78 |
| 5 | Phi(year)p(year) | 4 | 1172.513 | 2.865 | 0.064793 | 1164.454 |
| 6 | Phi(age + year)p(age) | 7 | 1173.098 | 3.450 | 0.048361 | 1158.931 |
| 7 | Phi(age + year)p(site) | 9 | 1173.149 | 3.501 | 0.047137 | 1154.881 |
| 8 | Phi(year)p(age) | 5 | 1173.933 | 4.285 | 0.031856 | 1163.844 |
| 9 | Phi(age)p(year) | 5 | 1173.953 | 4.305 | 0.031531 | 1163.864 |
| 10 | Phi(age + site + year)p(year) | 10 | 1174.206 | 4.558 | 0.027787 | 1153.877 |
| 11 | Phi( .)p(year) | 3 | 1174.625 | 4.976 | 0.022541 | 1168.589 |
| 12 | Phi(age + site + year)p(~site) | 13 | 1174.794 | 5.146 | 0.020713 | 1148.247 |
| 13 | Phi(age)p(.) | 4 | 1174.871 | 5.222 | 0.019933 | 306.4818 |
| 14 | Phi(year)p(site) | 7 | 1175.481 | 5.832 | 0.014693 | 1161.314 |
| 15 | Phi( .)p(.) | 2 | 1175.62 | 5.972 | 0.013702 | 311.2731 |
| 16 | Phi(age + site)p(year) | 9 | 1175.696 | 6.048 | 0.01319 | 1157.428 |
| 17 | Phi(age + site + year)p(age) | 11 | 1176.075 | 6.426 | 0.010917 | 1153.68 |
| 18 | Phi(year + site)p( .) | 7 | 1176.479 | 6.831 | 0.008919 | 1162.312 |
| 19 | Phi(age + site)p(.) | 8 | 1176.547 | 6.898 | 0.008622 | 300.0026 |
| 20 | Phi(year + site)p(year) | 8 | 1177.848 | 8.199 | 0.004499 | 1161.633 |
| 21 | Phi( .)p(age) | 4 | 1178.51 | 8.862 | 0.00323 | 310.1215 |
| 22 | Phi(age)p(age) | 6 | 1178.645 | 8.997 | 0.00302 | 306.1906 |
| 23 | Phi(age)p(site) | 8 | 1178.698 | 9.050 | 0.00294 | 302.1543 |
| 24 | Phi(site)p(year) | 7 | 1178.857 | 9.208 | 0.002716 | 1164.69 |
| 25 | Phi(age + site)p(site) | 12 | 1178.932 | 9.283 | 0.002617 | 294.1343 |
| 26 | Phi(year + site)p(age) | 9 | 1179.371 | 9.723 | 0.002101 | 1161.102 |
| 27 | Phi(site)p(.) | 6 | 1179.597 | 9.949 | 0.001876 | 307.1425 |
| 28 | Phi(.)p(site) | 6 | 1179.635 | 9.987 | 0.001841 | 307.1809 |
| 29 | Phi(year + site)p(site) | 11 | 1179.827 | 10.178 | 0.001673 | 1157.432 |
| 30 | Phi(age + site)p(age) | 10 | 1180.541 | 10.893 | 0.00117 | 299.8827 |
| 31 | Phi(age + site + year + site*year)p(.) | 13 | 1180.711 | 11.063 | 0.001075 | 1154.165 |
| 32 | Phi(site)p(site) | 10 | 1182.437 | 12.788 | 0.000454 | 301.7783 |
| 33 | Phi(site)p(age) | 8 | 1182.565 | 12.917 | 0.000425 | 306.0211 |
| 34 | Phi(age + site + year + site*year)p(year) | 14 | 1183.018 | 13.370 | 0.000339 | 1154.387 |
| 35 | Phi(age + site + year + site*year)p(site) | 17 | 1183.693 | 14.045 | 0.000242 | 1148.769 |
| 36 | Phi(age + site + year + site*year)p(age) | 15 | 1184.672 | 15.024 | 0.000148 | 1153.947 |

**Table S3**. Summary of parasitology results for each individual house sparrow caught during the study. (NA = appropriate sample not available, NEG = negative, POS = positive). See electronic version.

**Table S4.** Outputs of candidate models for investigating house sparrow survival in relation to *Plasmodium* intensity of infection, year and age of the individual birds. The best models are in bold. K= number of parameters, *=interaction, Plasm= *Plasmodium*. Phi=survival; p=recapture rate.

| **Model** | | **K** | **AICc** | **ΔAICc** | **AICc Weights** | **Deviance** |
| --- | --- | --- | --- | --- | --- | --- |
| 1 | **Phi(Plasm)p(.)** | 3 | 441.880 | 0.000 | 0.262 | 435.789 |
| 2 | **Phi(age*Plasm)p(.)** | 4 | 442.304 | 0.424 | 0.212 | 434.151 |
| 3 | **Phi(age + Plasm)p(.)** | 4 | 442.775 | 0.895 | 0.167 | 434.622 |
| 4 | **Phi(age + year + Plasm)p(.)** | 5 | 444.540 | 2.660 | 0.069 | 434.310 |
| 5 | **Phi(.)p(.)** | 2 | 444.916 | 3.036 | 0.057 | 199.132 |
| 6 | **Phi(age + year + Plasm + site)p(.)** | 9 | 445.419 | 3.538 | 0.045 | 426.715 |
| 7 | **Phi(age + Plasm + site)p(.)** | 8 | 445.795 | 3.915 | 0.037 | 429.235 |
| 8 | Phi(age + year + age*Plasm)p(.) | 6 | 445.914 | 4.033 | 0.035 | 433.589 |
| 9 | Phi(age + year + site + age*Plasm)p(.) | 10 | 446.246 | 4.366 | 0.030 | 425.383 |
| 10 | Phi(age)p(.) | 3 | 446.656 | 4.776 | 0.024 | 198.826 |
| 11 | Phi(age + year + site + site*Plasm)p(.) | 13 | 446.729 | 4.849 | 0.023 | 419.285 |
| 12 | Phi(year)p(.) | 3 | 446.781 | 4.901 | 0.023 | 198.951 |
| 13 | Phi(age + year)p(.) | 4 | 448.548 | 6.667 | 0.009 | 198.656 |
| 14 | Phi(site)p(.) | 6 | 449.597 | 7.716 | 0.006 | 195.534 |
| 15 | Phi(age + year + site)p(.) | 8 | 452.216 | 10.336 | 0.001 | 193.917 |

**Table S5.** Outputs of candidate models for investigating house sparrow survival in relation to *Atoxoplasma* intensity of infection, year, site and age of individual birds. The best models are in bold. K= number of parameters *=interaction, Atox= *Atoxoplasma*. Phi=survival; p=recapture rate.

| **Model** | | **K** | **AICc** | **ΔAICc** | **AICc Weights** | **Model Likelihood** |
| --- | --- | --- | --- | --- | --- | --- |
| 1 | **Phi(.)p(.)** | 2 | 441.465 | 0.000 | 0.348 | 197.268 |
| 2 | **Phi(year)p(.)** | 3 | 443.268 | 1.803 | 0.141 | 197.025 |
| 3 | **Phi(age)p(.)** | 3 | 443.339 | 1.874 | 0.136 | 197.096 |
| 4 | **Phi(Atox)p(.)** | 3 | 443.417 | 1.952 | 0.131 | 437.325 |
| 5 | **Phi(age + year)p(.)** | 4 | 445.168 | 3.703 | 0.055 | 196.863 |
| 6 | **Phi(age + Atox)p(.)** | 4 | 445.298 | 3.833 | 0.051 | 437.144 |
| 7 | Phi(age*Atox)p(.) | 4 | 445.475 | 4.010 | 0.047 | 437.321 |
| 8 | Phi(site)p(.) | 6 | 445.802 | 4.337 | 0.040 | 193.325 |
| 9 | Phi(age + year + Atox)p(.) | 5 | 447.174 | 5.709 | 0.020 | 436.942 |
| 10 | Phi(age + year + site)p(.) | 8 | 448.222 | 6.757 | 0.012 | 191.508 |
| 11 | Phi(age + year + age*Atox)p(.) | 6 | 449.206 | 7.741 | 0.007 | 436.881 |
| 12 | Phi(age + Atox +site)p(.) | 8 | 449.887 | 8.422 | 0.005 | 433.325 |
| 13 | Phi(age + year + Atox + site)p(.) | 9 | 450.349 | 8.884 | 0.004 | 431.643 |
| 14 | Phi(age + year + site + age*Atox)p(.) | 10 | 452.368 | 10.902 | 0.001 | 431.501 |
| 15 | Phi(age + year + site + site*Atox)p(.) | 13 | 453.814 | 12.349 | 0.001 | 426.364 |

**Table S6**. Result of a possible association between environmental NO_2_ and house sparrow population growth rate at the 11 study sites. Estimates ± standard error (s.e) of the interaction between year and pollution are reported, alongside the F test and its significance.

| **Interaction estimates**  **± s.e** | **F** | **P** |
| --- | --- | --- |
| 0.006±0.015 | 0.184 | 0.669 |
